# A lightweight co-optimization model for field sunflower disease identification

**DOI:** 10.3389/fpls.2025.1728123

**Published:** 2026-01-05

**Authors:** Xiao Wu, Liqian Zhang, Yaogeng Wang, Yunli Bai

**Affiliations:** 1 College of Computer and Information Engineering, Inner Mongolia Agriculture University, Hohhot, China; 2 Inner Mongolia Autonomous Region Key Laboratory of Big Data Research and Application of Agriculture and Animal Husbandry, Hohhot, China

**Keywords:** deep learning, lightweight, raspberry pi, sunflower disease recognition, YOLO

## Abstract

**Introduction:**

Accurate identification of crop diseases is crucial for ensuring crop quality and yield. However, existing deep learning models for crop disease identification lack robustness in complex field environments and suffer from large model parameter sizes, which makes them difficult to deploy on resource-constrained devices. This gap between laboratory models and practical field applications necessitates the development of a lightweight and robust identification model.

**Methods:**

To address these challenges, this paper proposes a lightweight YOLO-CGA model for sunflower disease identification and deploys it on a Raspberry Pi for field application. The model incorporates three key improvements based on YOLOv8n-cls: (1) A CBAM_ADown module is designed, which integrates attention mechanisms with asymmetric downsampling to enhance feature extraction and noise suppression in complex image backgrounds; (2) The C2f module of YOLOv8n-cls is replaced with the C3Ghost module, which utilizes ghost convolution to reduce parameter count while preserving fine-grained features; (3) An AFC_SPPF module is constructed, which aggregates multi-scale disease features through a multi-branch adaptive fusion structure to improve recognition performance for diverse lesions.

**Results:**

Experimental results on three major datasets show that the proposed YOLO-CGA model achieves high identification accuracy: 98.48% on the BARI-Sunflower dataset, 98.32% on the Cotton Disease Dataset, and 91.11% on the FGVC8 dataset. Meanwhile, the model maintains a lightweight property with only 0.92M parameters, which is significantly fewer than that of other comparative models.

**Discussion:**

The deployment of the YOLO-CGA model on the Raspberry Pi end device effectively bridges the gap between laboratory models and field applications, fulfilling the demand for real-time and on-site crop disease identification. The integration of attention mechanisms, ghost convolution, and multi-scale feature fusion enables the model to balance accuracy, robustness, and lightweight performance, making it suitable for resource-limited field scenarios.

## 1 Introduction

Sunflower is one of the most important crops of global agriculture and is an important source of edible oil and fodder. As the third largest oilseed crop in the world (Pilorgé, 2020), sunflower accounts for more than 12% of the global consumption of vegetable oils. Beyond their economic value, sunflowers can absorb heavy metal pollutants such as lead and cadmium from the soil through their root systems, effectively reducing soil contamination levels (Alaboudi et al., 2018). However, these important crops are chronically threatened by fungal, bacterial and viral pathogens. The major diseases affecting the yield of sunflowers are downy mildew (Qi et al., 2023), gray mold (Tomioka and Sato, 2011), sclerotinia rot (Sui et al., 2025) and rust (Berghuis et al., 2022). Sclerotinia rot is one of the primary diseases affecting sunflower crops, causing an average yield reduction of 10%-20% (Fusari et al., 2012). Therefore, timely and accurate prediction of sunflower diseases and precise control measures can significantly reduce the impacts caused by sunflower diseases.

Traditional methods of plant disease identification rely mainly on manual observation, which are inefficient and inaccurate. With the development of deep learning, many researchers have combined deep learning with agriculture (Vinod Chandra et al., 2024) to achieve timely and accurate identification of plant diseases. Earlier, some classical CNN architecture models such as AlexNet (Li et al., 2022), GoogleNet (Indrawati et al., 2023), VGGNet (Sanida et al., 2023), ResNet (Ding et al., 2022), and DenseNet (Nandhini and Ashokkumar, 2022) were commonly used as base models for plant disease recognition.

In addition to the standard CNN architecture models for plant disease recognition, there are other models that have achieved good results in plant disease recognition. Feng et al. (2024) proposed an EDIT model architecture based on Vision Transformer. On three datasets (namely PlantVillage, FGVC8, and EMBRAPA), this model achieved accuracies of 99.9%, 91.5%, and 97.4%, respectively. Zhang et al. (2025) constructed a DDHTL-VMamba model by combining VMamba, Diffusion Modelling, and Transfer Learning. This model achieved an accuracy of 99.81% on the PlantVillage dataset. Wang et al. (2022) proposed a model based on YOLOv5, which achieved an accuracy of 90.26% on the PlantDoc dataset. This model incorporates optimization strategies such as an improved attention mechanism, GhostNet, and a weighted bidirectional feature pyramid network (BiFPN). Liu and Zhang (2023) proposed a PiTLiD model by combining the pre-trained Inception-V3 with transfer learning technique, and this model achieved an accuracy of 98.2% on a self-constructed small sample dataset.

Although many of the above studies have made significant breakthroughs in the core performance of plant disease identification, they are also accompanied by a series of growing problems. Specifically, model architectures have become increasingly complex, and the number of parameters has risen significantly. Meanwhile, drawbacks such as the difficulty of deploying on resource-constrained edge devices, high computational costs, and poor real-time performance have grown increasingly prominent. For these reasons, many researchers have begun to focus on the study of lightweight models. Li et al. (2023) proposed a PWVT lightweight model with only 98M parameters and achieved 93.6% accuracy on the wheat dataset. Yang et al. (2023) proposed a lightweight DGLNet network model based on the attention mechanism and dynamic convolution. This model achieved accuracies of 99.82% and 99.71% on two datasets, namely PlantVillage and Paddy Doctor. Ji et al. (2024) proposed a lightweight model, ICS-ResNet, which was based on the ResNet50 lightweight network and combined with improved spatial and channel attention modules as well as a depth-separable residual structure. This model not only significantly improved the accuracy of maize leaf disease recognition but also reduced the number of parameters and computation by 69.12% and 54.88%, respectively. Gole et al. (2023) proposed a lightweight TrlncNet model by replacing the MLP block in Vision Transformer with a customized Inception block. Compared to the Vision Transformer, this model achieved accuracy improvement of 5.38% and 2.87% on the PlantVillage and Maize datasets, respectively. Although the above studies have made significant progress in model lightweighting, the field of crop disease recognition still faces many challenges. In complex natural field environments, disease identification is often constrained by multiple factors such as background interference, harmful weather effects, and leaf shading. Therefore, constructing robust lightweight models that can adapt to complex field contexts and deploying them in resource-constrained devices to land in real agricultural scenarios remains an urgent challenge.

Specifically, sunflowers grow in complex natural field environments. Key disease features are often obscured by various factors. These factors include background interferences like soil and weeds, adverse weather such as rain and fog, and leaf occlusion. As a result, critical characteristics such as the white mildew layer of downy mildew and the spore piles of rust are hard to distinguish. This puts existing models in a core “performance-efficiency” dilemma. If a model is built to be robust in complex scenarios, it tends to be too large. Thus, it cannot be deployed on low-cost edge devices like Raspberry Pi. If parameters are simply reduced for lightweight, important feature details will be lost. Then, the recognition accuracy in actual fields cannot be guaranteed. This dilemma directly limits the application of related technologies in on-site real-time detection for farmers. Therefore, developing a robust and lightweight model becomes the core motivation of our YOLO-CGA model’s architecture design. This model should both adapt to complex field environments and be deployable on edge devices. It also serves as a targeted solution to the limitations of previous studies.

To solve this dilemma, this study designs the lightweight YOLO-CGA model, using YOLOv8n-cls as the base model. First, to address the issue of “field background noise obscuring features”, we propose the CBAM_ADown module. This module combines the CBAM attention mechanism with asymmetric downsampling (ADown). It strengthens channels with disease features and weakens those with soil background through channel weight assignment. Meanwhile, it uses spatial attention to focus on tiny lesions. With the help of asymmetric downsampling, it preserves fine-grained details like the spore pile texture of rust. In this way, it achieves the dual goals of “noise filtering + feature enhancement”. Second, to balance lightweight design and feature integrity, we replace the C2f module of YOLOv8n-cls with the C3ghost module. Ghost convolution has the characteristic of “generating features through low-cost linear transformation”, and this replacement reduces parameters by 35.84% without losing key disease information. Finally, to handle the scale variation of field lesions (from millimeters to centimeters), we design the AFC_SPPF module with multi-branch adaptive fusion. It combines branches of multi-pooling and identity mapping to capture multi-scale features. This enhances the model’s ability to recognize complex lesions, such as compound diseases and edge-blurred diseases.

These three modules form a collaborative closed-loop, following the logic of “feature purification, lightweight feature transmission, and multi-scale feature complementation”. Eventually, they achieve the optimal balance between “performance and efficiency”. The YOLO-CGA model reaches a recognition accuracy of 98.48% with only 0.92M parameters. Compared with the baseline model YOLOv8n-cls, it reduces parameters by 36.6% and GFLOPs (computational load) by 58.8%. Meanwhile, its average accuracy increases by 2.96%. Currently, the model has been successfully deployed on Raspberry Pi edge devices. It enables real-time on-site disease detection in agricultural scenarios and effectively addresses farmers’ practical needs. Based on these achievements, the main contributions of this study are as follows: First, we proposed the CBAM_ADown module, which provides a new idea for crop disease feature purification under complex field backgrounds. Second, we designed an AFC_SPPF module and verified the collaborative optimization value of CBAM_ADown, C3ghost, and AFC_SPPF modules, offering references for module design of similar models. Third, we developed the YOLO-CGA model with both lightweight and high-precision advantages. Its successful deployment on Raspberry Pi provides a practical solution for the application of agricultural disease recognition technology.

## 2 Materials and method

### 2.1 Data pre-processing and evaluation indicators

#### 2.1.1 Acquisition of datasets

**Figure 1:**
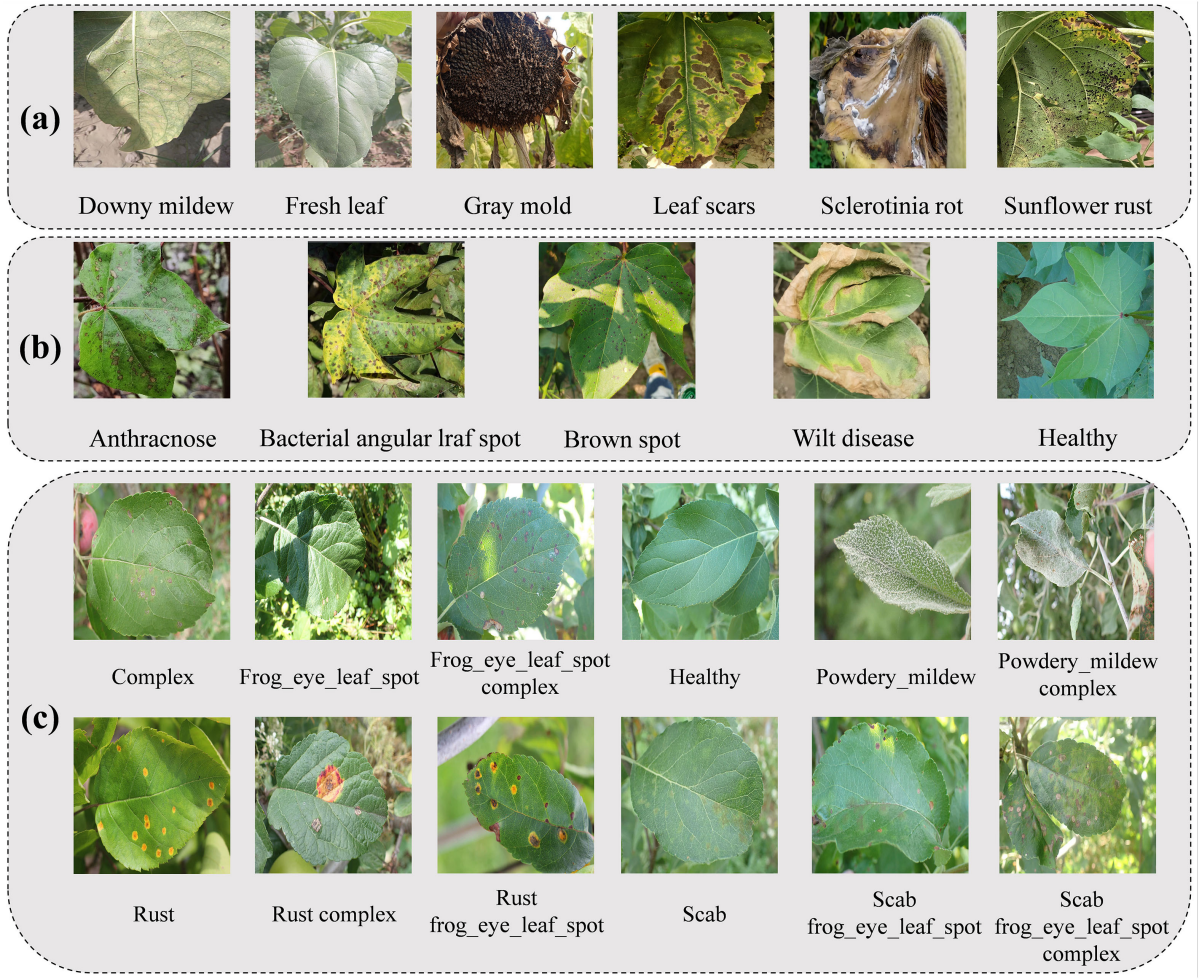
Images of plant disease datasets in three complex natural field contexts. Among them, **(a)** is the BARI-Sunflower dataset, **(b)** is the Cotton Disease Dataset, and **(c)** is the FGVC8 dataset.

The dataset used in this study is from the publicly available sunflower image dataset BARI-Sunflower (Sara et al., 2022), which contains 466 original images and 1892 enhanced images. These images contain leaves, stems, and corollas of diseased sunflowers as well as leaves of healthy sunflowers, and the resolution of the images is 4000 × 3000. We only used the original images of this dataset. These original images contain four categories, namely Downy mildew, Gray mold, Leaf scars, and Fresh leaf. In addition, we collected 65 sample images of Sclerotinia rot and 305 sample images of Sunflower rust from the Web. These newly collected images were combined with the original images from the BARI-Sunflower dataset. Together, they form the original dataset we used for the sunflower disease identification task, as shown in **Figure 1A**.

To validate the generalization ability of our proposed model, we used two public datasets, which are the Cotton Disease Dataset (Zhao et al., 2023) and the FGVC8 (Kaggle, 2021). They are both constructed based on complex natural field contexts. The Cotton Disease Dataset contains 3033 original images. These images, with a resolution of 638 × 854, contain leaves of diseased and healthy cotton, which are classified into five categories, namely, Anthracnose, Bacterial angular leaf spot, Brown spot, Healthy, and Wilt disease, as shown in **Figure 1B**. The FGVC8 dataset contains a total of 18632 images, which include images of diseased and healthy apple leaves, and the resolution of the images is 4000 × 2672. These images are classified into six major categories: Complex, Frog eye leaf spot, Healthy, Powdery mildew, Rust, and Scab. These are further subdivided into twelve subcategories, as shown in **Figure 1C**.

A detailed descriptions of the sample information for each category of the above three datasets are shown in **Table 1**.

**Table 1:** Detailed descriptions of samples in each category of the three datasets.

Dataset	Classes	Numbers
BARI-Sunflower	Downy mildew	120
	Gray mold	72
	Leaf scars	72
	Fresh leaf	134
	Sclerotinia rot	65
	Sunflower rust	305
Cotton disease dataset	Anthracnose	816
	Bacterial angular leaf spot	501
	Brown spot	916
	Healthy	263
	Wilt disease	537
FGVC8	Complex	1602
	Frog_eye_leaf_spot	3181
	Frog_eye_leaf_spot complex	165
	Healthy	4624
	Powdery_mildew	1184
	Powdery_mildew complex	87
	Rust	1860
	Rust complex	97
	Rust frog_eye_leaf_spot	120
	Scab	4826
	Scab frog_eye_leaf_spot	686
	Scab frog_eye_leaf_spot complex	200

#### 2.1.2 Data augmentation

In this study, all three datasets went through a unified data preprocessing process. First, all images were uniformly resized to 512×512 pixels to minimize information loss and enhance training efficiency and stability. Subsequently, the datasets were expanded using three data augmentation methods.

**Figure 2:**
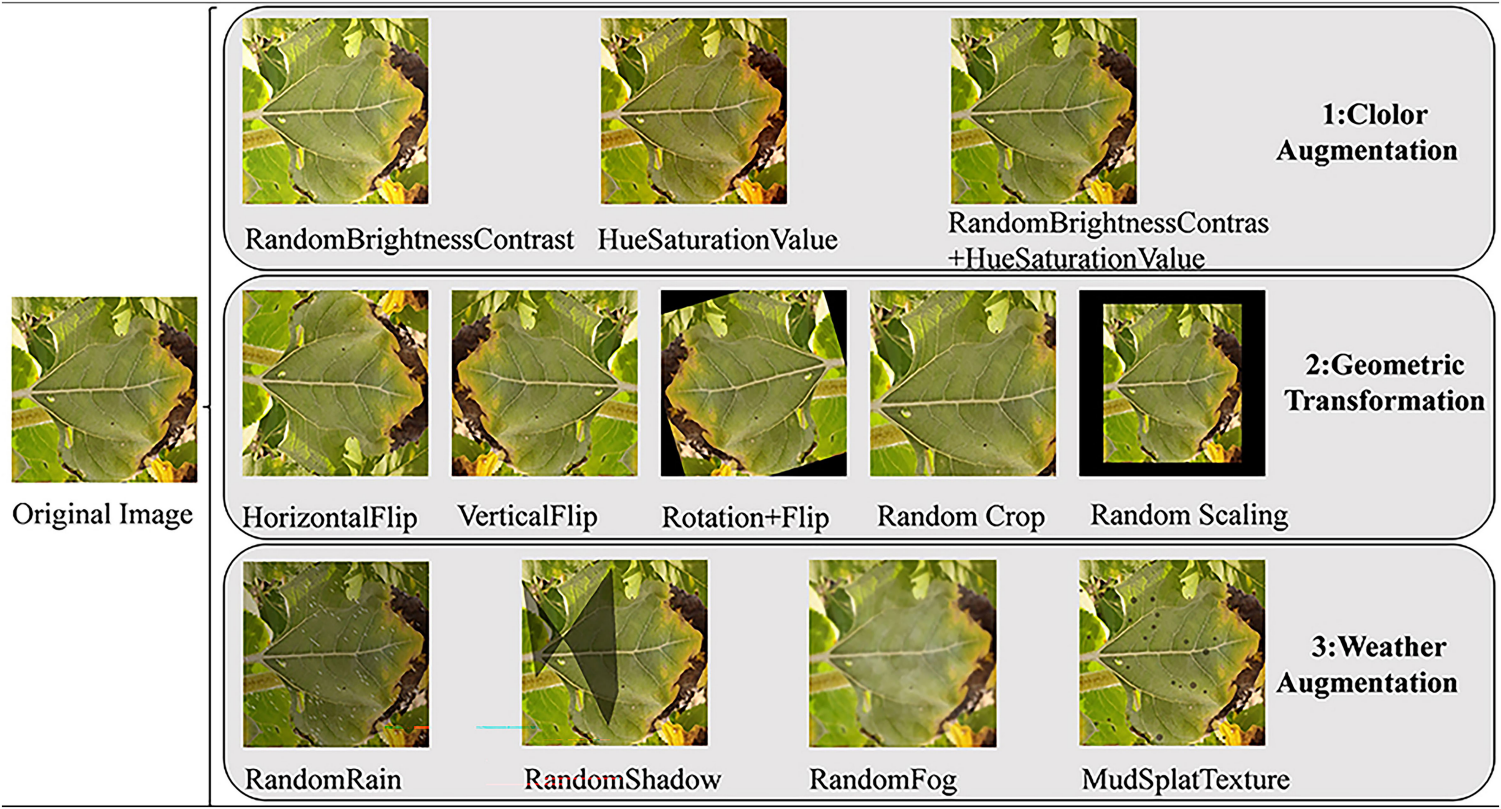
Plant disease data augmentation effect diagram.

The first data augmentation mode is color augmentation. Lighting and color variations in complex natural fields were simulated by adjusting the images’ random brightness and contrast, random Hue and saturation, and a combination of the two methods. The adjustment range for random brightness and contrast was ±20% of the original data, and the adjustment range for random Hue and saturation was ±15% of the original image. The second data augmentation mode is a geometric transformation. The diversity of spatial positions of the image was extended by simulating different photo angles through horizontal flipping, vertical flipping, rotating and combining flipping, translation transformation, scaling transformation, and random cropping. The third data augmentation method is weather augmentation (Dai et al., 2023). A variety of different weather often accompanies complex natural fields. In order to more realistically simulate the complex natural field background, enhance the robustness of the model to complex environments and improve its anti-jamming ability, we used four weather enhancement techniques. We used random raindrops to simulate real rainy weather by setting the type of raindrops to drizzle, the length of the raindrops to 6 pixels, the width of the raindrops to 1 pixel, the blurring level to 1, and reducing the brightness factor to 70% of the original image. Random shadows are used to simulate the effect of shadows created by leaves in light, setting the shadow coverage to the entire image and the dimension size to 6. A stochastic fog transformation was used to simulate the effect of fog on natural fields, setting the transparency coefficient of fog to 0.05. Mud splashes were used to simulate the effect of mud spots on plant leaves in a natural field by setting the density of mud spots to be 0.0001, i.e., every 10000 pixels contains a mud spot, and the radius of the mud spot is a random 5–10 pixels. Each selected data augmentation method was applied with a probability of 1.0, meaning it is always executed once chosen. The data augmentation process is illustrated in **Figure 2**.

**Figure 3:**
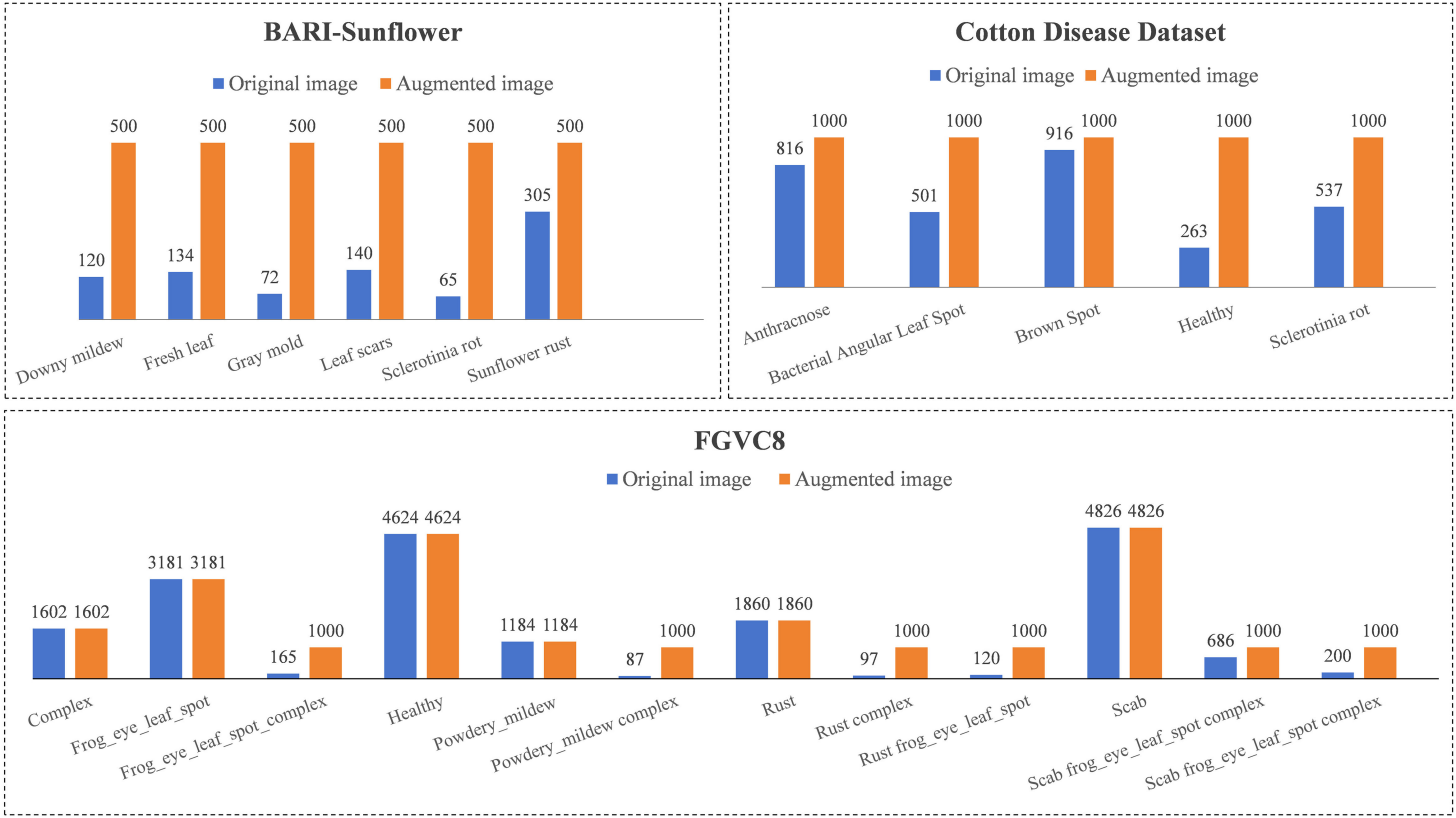
Comparison of samples counts in each category before and after data enhancement for the three datasets.

All three datasets we used are imbalance in category. During the training process, the imbalance causes the model to focus more on the features of leaf disease images from categories with a larger number of samples. At the same time, it also makes the model less robust to leaf diseases from categories with a smaller number of samples. For BARI-Sunflower, we augmented the image data to 500 images per category. For the cotton disease dataset, we augmented the image data to 1,000 images per category. For FGVC8, we augmented the image data to 1,000 images for the categories with a number of samples below 1,000. The comparison graph of the number of samples in each category of the three datasets before and after using data augmentation is shown in **Figure 3**.

#### 2.1.3 Model evaluation indices and experimental parameter settings

In this study, in order to verify the reliability of our proposed model, we need to use the same hardware, software, hyperparameters, and scientific evaluation methods to evaluate our model. The experimental environment for this study was configured as follows: operating system Windows 11; GPU server V100S (with CUDA 11.8), Python version 3.8.20, and PyTorch version 2.0. The hyperparameter settings for all experiments in this study are shown in **Table 2**.

**Table 2:** Hyperparameters setting of the model training.

Hyperparameters	Value
Image size	512×512
Batch size	32
Epoch	100
Patience	0
workers	8
Optimizer	Adam
Lr0	0.001
Lrf	0.01
Momentum	0.937
Weight_dacay	0.0005
Seed	42

We have used the idea of K-fold cross-validation (Kohavi, 1995) to evaluate the performance of our proposed model. In this study, the value of K is taken as 5. The dataset is divided into a training set and an independent test set according to 8:2. Five-fold cross-validation is performed on the training set, and each fold further splits the training set into a sub-training set and a validation set in 8:2. In each fold of training, the weights that performed best on the validation set are retained. Afterwards, the five sets of optimal weights are separately evaluated on the test set. Finally, the average values of accuracy, precision, recall, and F1-score are used as the core indicator to measure the model’s performance in the sunflower disease image classification task, while the standard deviations of these metrics serve as a measure of the model’s stability.

(1) $$\text{Accuracy}=\frac{TP+TN}{TP+TN+FP+FN}$$

(2) $$\text{Precision}=\frac{TP}{TP+FP}$$

(3) $$\text{Recall}=\frac{TP}{TP+FN}$$

(4) $$\text{F}1=2\times \frac{\text{Precision}\times \text{Recall}}{\text{Precision+Recall}}$$

In Equations 1–4, TP is the number of samples correctly predicted to be in the positive category, TN is the number of samples correctly predicted to be in the negative category, FP is the number of samples incorrectly predicted to be in the positive category, and FN is the number of samples incorrectly predicted to be in the negative category.

### 2.2 The YOLO-CGA model architecture

YOLO series models are popular among many researchers as a real-time and high-precision target detection models. With the iterative updating of versions, their functions have been expanded to multiple tasks such as image classification, semantic segmentation, pose estimation, etc. The main structure of the YOLO series model consists of three main parts: Backbone, Neck, and Head. Backbone is responsible for extracting the basic features from the input image. Neck fuses and enhances the features extracted by Backbone, and connects low-level features with high semantic information. Head is used to output the final result. The model architecture for classification in the YOLO series inherits the Backbone from the detection model and replaces the Head part with a classification module.

**Figure 4:**
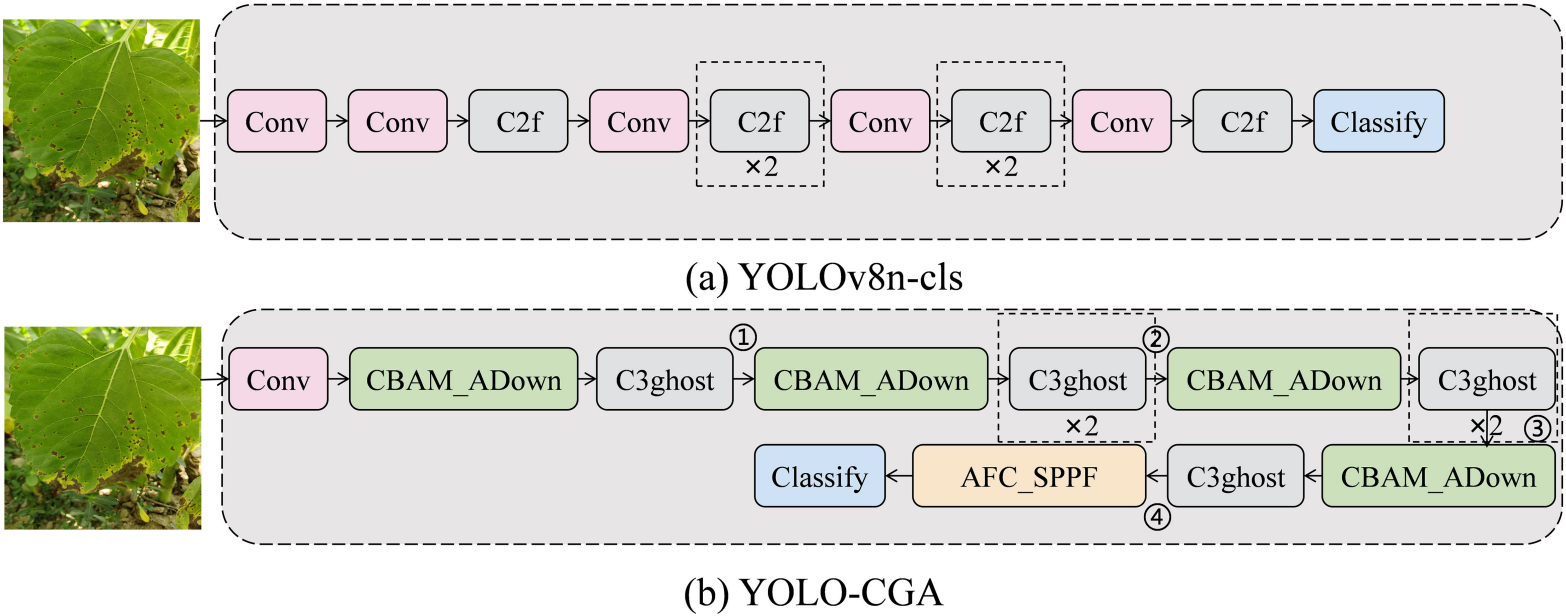
Comparison of the structural diagrams of the YOLOv8n-cls **(a)** and YOLO-CGA **(b)** models. Where ①, ②, ③, and ④ in **(b)** will be explained in the section 3.3.

Using the lightweight YOLOv8n-cls (Yaseen, 2024) classification model of YOLOv8n-cls as our base model, we proposed a lightweight model called YOLO-CGA. The model aims to solve the problem of insufficient accuracy in sunflower disease identification in complex field contexts and the difficulty in deploying the model in resource-constrained devices. The differences between the proposed model and YOLOv8n-cls are shown in **Figure 4**. The CBAM_ADown module is used in the YOLO-CGA model instead of the standard convolutional module CBS to purify the disease feature information and suppress unneeded background noise. In addition, the C3ghost module replaces the C2f module to drastically reduce the number of parameters in the model and generate more fine-grained information. Finally, a new AFC_SPPF module is introduced to obtain features of sunflower diseases at different scales.

#### 2.2.1 CBAM_ADown module

Asymmetric Downsampling (ADown) was first proposed in YOLOv9 (Wang et al., 2025), replacing part of the convolutional downsampling module due to its smaller number of covariates and stronger feature extraction capability. Convolutional downsampling reduces the size of the feature map by sliding the convolutional kernel, but at the same time, it loses high-frequency detail information, such as the edge of the lesion and texture information. Pooling downsampling retains only the maximum or average value, resulting in reduced feature expression and inadequate recognition of small targeted areas of disease spots. ADown enhances feature extraction capabilities through a parallel dual-branch architecture, leveraging complementary operations of convolution downsampling and pooling downsampling. Although ADown is more capable of feature extraction compared to normal convolutional downsampling, the feature extraction capability for plant diseases in complex field contexts is still limited. In sunflower disease images, disease features are often deeply intermixed with channel information from complex backgrounds. ADown accomplishes downsampling through simple two-branch parallel fusion, which lacks a targeted attention mechanism to differentiate key disease features from irrelevant background noise.

**Figure 5:**
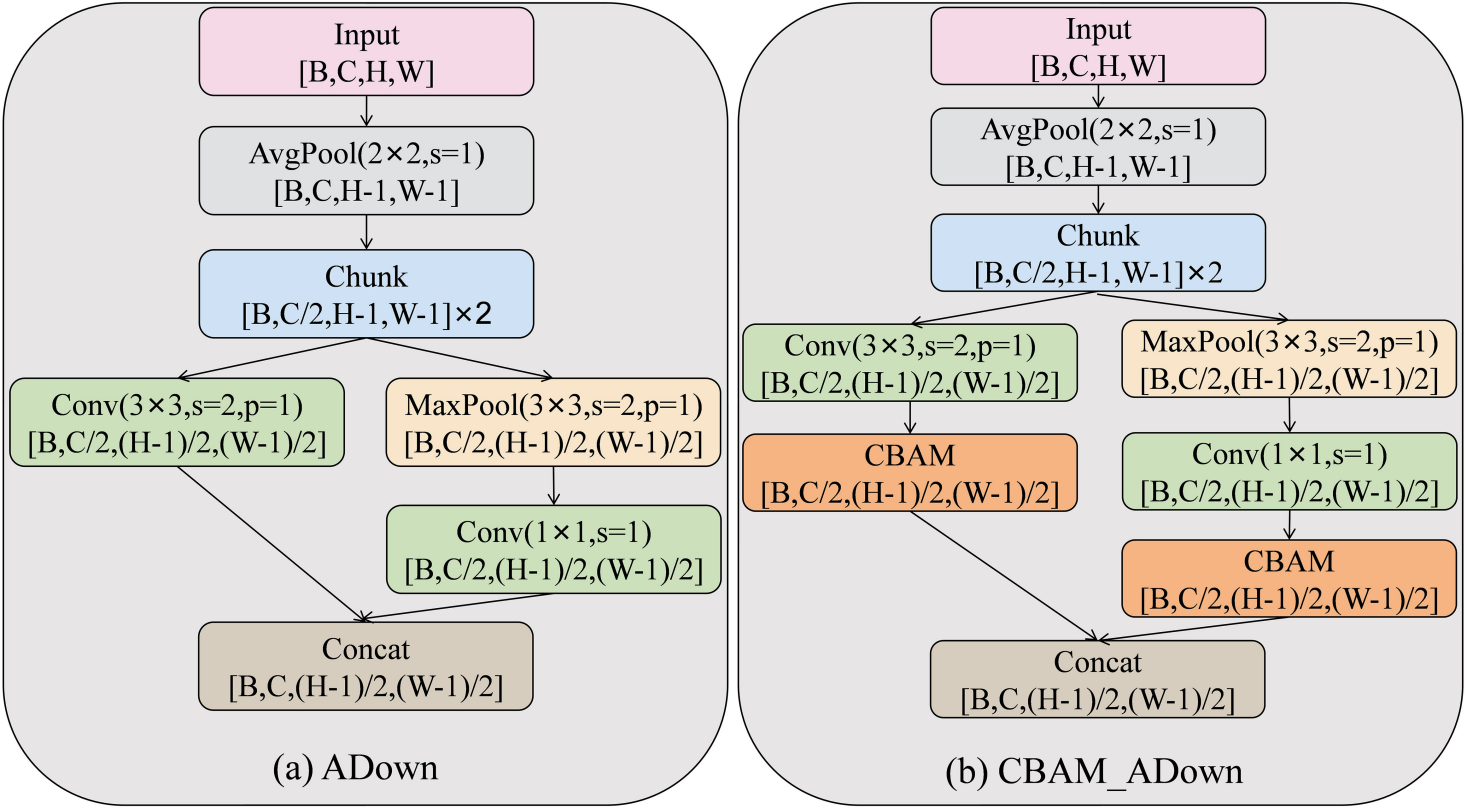
Structure of the ADown **(a)** and CBAM_ADown **(b)** modules.

Therefore, this paper proposes the CBAM_ADown module, and the structure of the module is shown in **Figure 5**. This module organically combines the parallel two-branch structure of ADown and Convolutional Block Attention Module (CBAM) (Woo et al., 2018). Its goal is to realize the accurate enhancement of disease features and the dynamic suppression of background noise. Through this combination, the module further enhances the ability of feature purification under a complex background. The channel attention mechanism is utilized to learn the channel response differences between disease features and background information. Specifically, it adaptively assigns higher weights to channels with key disease information. At the same time, it attenuates the weights of redundant information channels. These redundant channels include healthy leaf green channels and soil background channels. This targeted adjustment effectively solves the problem of overlapping channel information. Spatial attention mechanism generates spatial weights based on local texture and positional features of diseased areas, focusing on tiny spots and suppressing weights for large healthy leaves and irrelevant background areas. Through the synergistic integration of an attention mechanism and a dual-branch downsampling structure, CBAM_ADown retains the advantages of the original ADown module. Specifically, these advantages include low parameter counts and complementary feature representation. Furthermore, it significantly improves the model’s ability to highlight key lesion regions in sunflower diseases in complex backgrounds. Additionally, it enhances suppression of irrelevant background information, leading to more focused and accurate feature extraction.

#### 2.2.2 C3ghost module

**Figure 6:**
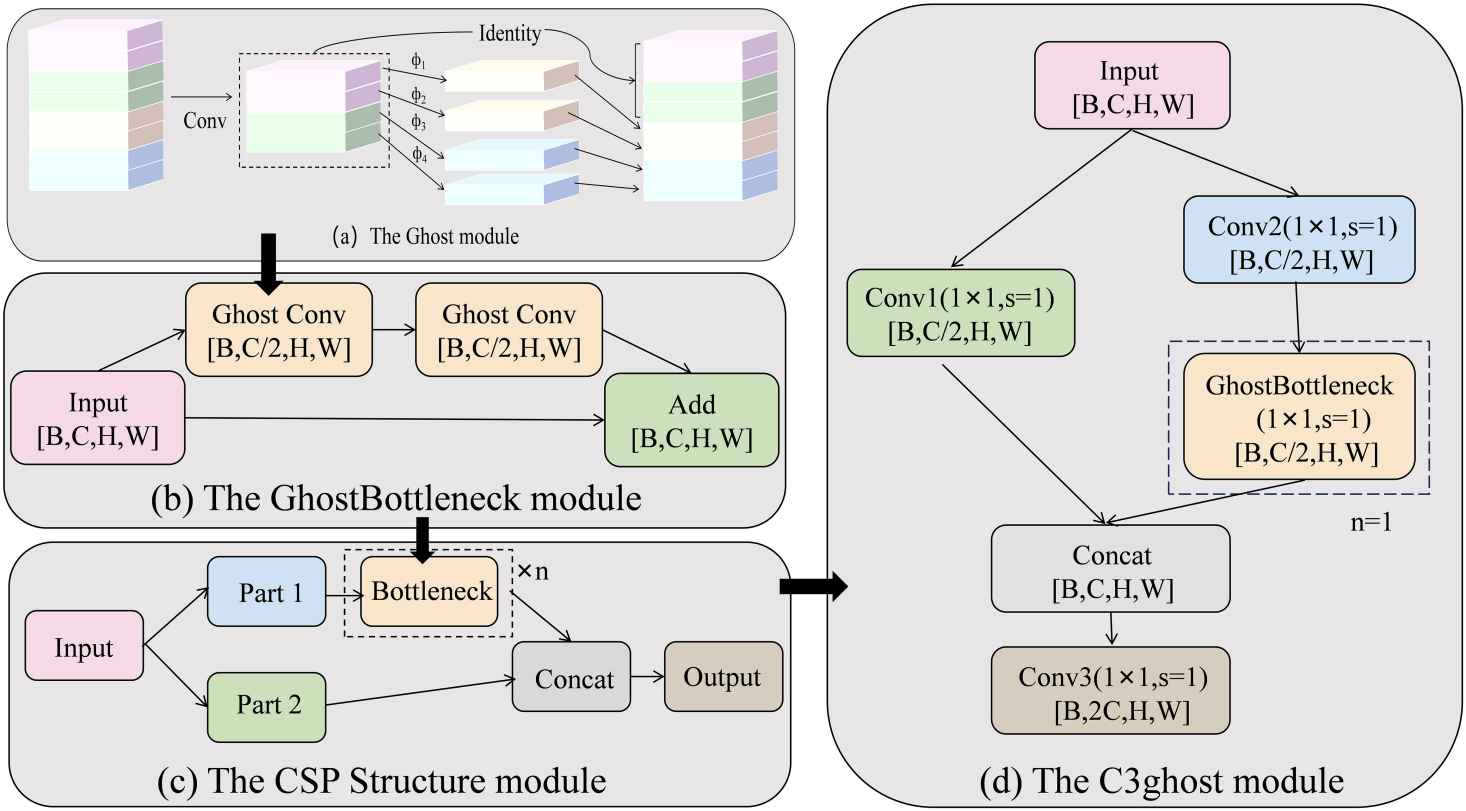
Structure of the C3ghost module. Among them, **(a)** is the Ghost module, **(b)** is the GhostBottleneck module, **(c)** is the CSP Structure module, and **(d)** is the C3Ghost module.

The Cross Stage Partial (CSP) structure was proposed by Wang et al. (2020a) in 2019. This architecture splits input features into two components along the channels: one undergoes deep feature extraction via the main path (incorporating multiple convolutional or residual blocks), while the other preserves raw information directly through the segment path. The CSP architecture effectively resolves computational redundancy and gradient repetition issues in CNNs, while simultaneously achieving both feature diversity and computational efficiency. It has become a core backbone component in models such as the YOLO series. Ghost Convolution originates from GhostNet (Han et al., 2020), which was proposed at CVPR 2020 and generates more fine-grained features with lower parameter counts through low-cost operations. The combination of the two forms the C3ghost module, which retains the advantage of cross-stage feature fusion of the CSP structure and further reduces redundant computation by replacing the traditional convolution with Ghost convolution. The structure of the C3ghost module is shown in **Figure 6**.

#### 2.2.3 AFC_SPPF module

**Figure 7:**
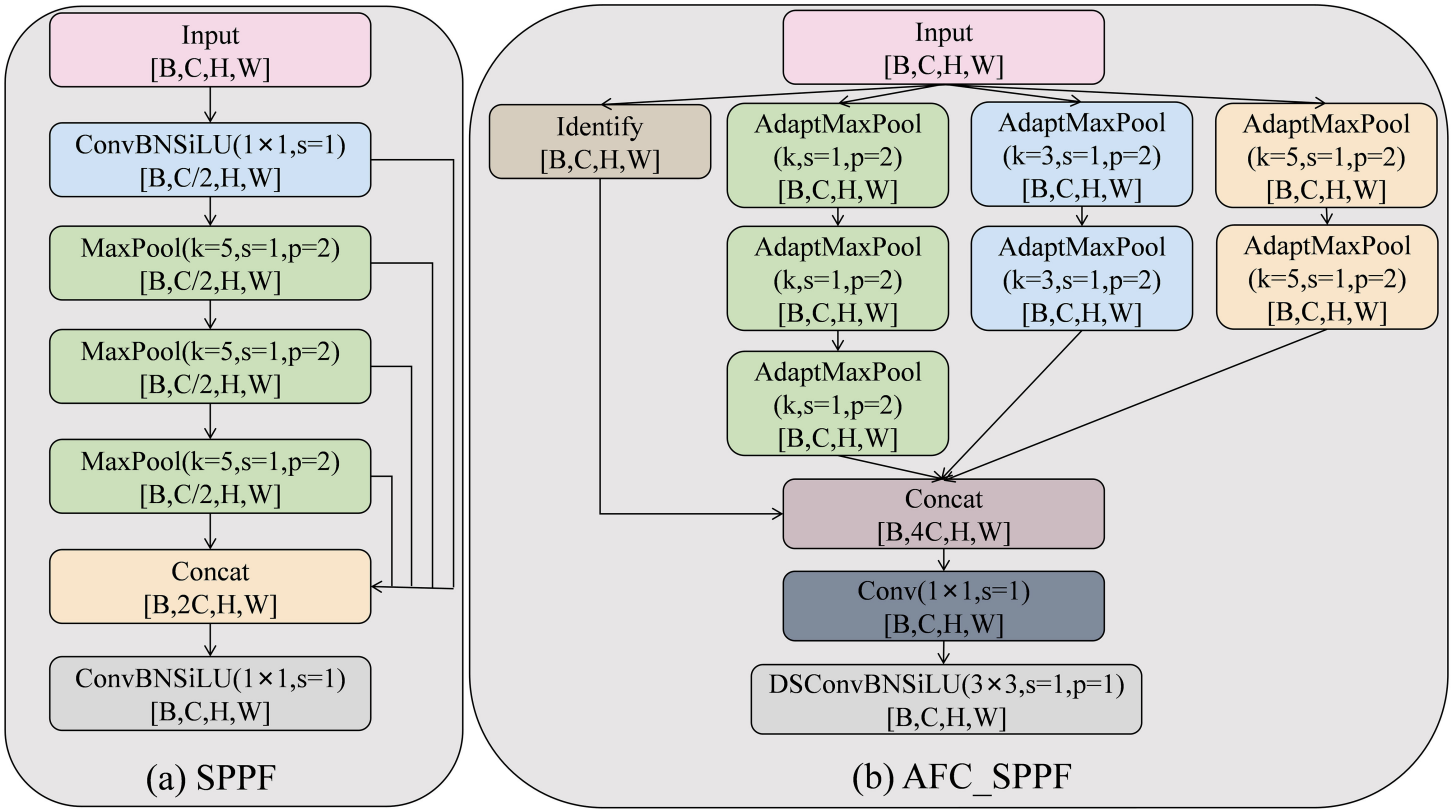
Structure of the SPPF **(a)** and AFC_SPPF **(b)** modules.

As a computationally efficient variant of the SPP (He et al., 2015) module, the SPPF (Ultralytics, 2025) module achieves a significant reduction in complexity by substituting the original multi-parallel large-kernel pooling with a more efficient cascade of small-kernel operations. SPPF has a fixed single pooling path, which limits the diversity of feature expression and make it prone to losing critical fine-grained feature information in the face of complex background interference. As shown in **Figure 7**, we propose an innovative module, the Adaptive Feature Concatenation SPPF module (AFC_SPPF). This module overcomes the limitations of a single pooled path by building a multi-branch parallel architecture. AFC_SPPF is designed with four parallel branches: the identity mapping branch retains the original features; the main branch performs multilevel pooling feature extraction; and the two auxiliary branches use pooling operations with different kernel sizes (3×3 and 5×5) to capture feature information from different sensory fields. The output size is ensured to be consistent through an adaptive pooling mechanism, and finally, the effective fusion of multi-scale features is achieved through feature splicing and channel compression. AFC_SPPF exhibits the following advantages: (1) The multi-branch parallel structure can simultaneously capture the detailed features and contextual information of the disease, improving the detection ability of disease spots at various scales. (2) The adaptive feature fusion mechanism enhances the robustness of the model to complex background interference, effectively distinguishing the disease region from the background noise. (3) The preservation of the identity mapping branch avoids the excessive loss of feature information, ensuring that the original disease features are transmitted completely. (4) The feature expression capability is further enhanced by the post-processing of deep separable convolution and batch normalization, providing a richer feature representation for accurately identifying crop disease types.

## 3 Experimental results and analysis

In this section, we first analyze the contribution of each core module (CBAM_ADown, C3ghost, AFC_SPPF) to the performance of the proposed model through ablation experiments. The effectiveness of individual components and the collaborative optimization mechanism between components was clarified. In particular, emphasis was placed on validating the feature extraction capability of the innovative CBAM_ADown module and the multiscale fusion value of the AFC_SPPF module. Second, on the BARI-Sunflower dataset, the proposed YOLO-CGA model is comprehensively analyzed in comparison with the benchmark model (YOLOv8n-cls) and mainstream lightweight models (e.g., MobileNetV3-Small, GhostNetV2_0.5, etc.). The proposed model’s advantages in balancing accuracy and efficiency were systematically evaluated through validation across two dimensions. These dimensions include lightweight metrics such as the number of parameters and GFLOPs, as well as recognition performance metrics, including average accuracy and standard deviation. Finally, to further validate the generalization performance of the model, generalization experiments are carried out on two cross-crop public datasets: Cotton disease dataset and FGVC8. These experiments aim to verify the proposed model’s adaptability to different crops, diverse disease types, and complex field contexts. 

### 3.1 Ablation experiments

In order to verify the mutual synergistic effectiveness of the modules, we conducted a series of ablation experiments using the BARI-Sunflower dataset based on the benchmark model YOLOv8n-cls. The ablation experiments for each module are shown in **Table 3**.

**Table 3:** Results of the ablation experiments on the proposed modules.

# of experiment	CBAM_ADown	C3ghost	AFC_SPPF	Parameters	GFLOPs	Average accuracy ± standard deviation (%)
0				1,445,974	3.4	95.52 ± 0.89
1	✓			1,169,378	2.7	97.95 ± 0.20
2		✓		927,778	1.9	95.45 ± 0.28
3			~	1,711,446	3.6	97.88 ± 0.56
4	✓	✓		651,122	1.2	97.88 ± 0.20
5	✓		~	1,434,790	2.9	98.50 ± 0.31
6		✓	~	1,196,250	2.1	97.95 ± 0.42
7	✓	✓	~	916,594	1.4	98.48 ± 0.38

Where “√” means replace the module in the model with the module, and “~” means add the module to the model.

As shown in **Table 3**, Experiment 1 uses the CBAM_ADown module to replace the CBS module in the baseline model: the number of parameters and GFLOPs of the model decreased by 19.56% and 20.59%, respectively, and the average accuracy increased by 2.43%. This result shows the CBAM_ADown inherits the advantages of the ADown module, including its low the number of parameter and feature complementarity. Moreover, it further enhances two key capabilities: the ability to extract key disease features, and the ability to suppress complex background noise. These enhancements are achieved through the synergistic effect of the channel and spatial attention mechanisms. Experiment 2 replaces the C2f module in the benchmark model with the C3ghost module. This replacement leads to a significant decrease in both the number of parameters and GFLOPs: the former drops by 35.84%, while the latter falls by 44.12%. At the same time, the average accuracy decreases slightly by 0.07%, and this decrease is negligible. This fully validates the lightweight advantage of the C3ghost module in achieving a significant increase in feature extraction efficiency while maintaining a largely stable performance. Experiment 3 introduces the innovative AFC_SPPF module in the baseline model to further enhance the multi-scale representation of features. The experimental results show that the introduction of the AFC_SPPF module improves the average accuracy by 2.36%, despite the fact that the number of parameters and GFLOPs of the model increase by 18.36% and 5.88%, respectively. Based on the respective advantages of the above three modules, we further synergistically integrated the CBAM_ADown, C3ghost and AFC_SPPF modules as demonstrated in Experiments 4, 5, 6, and 7. This integration ultimately constitutes YOLO-CGA, which is a lightweight classification model that incorporates the advantages of multiple modules. The number of parameters and computations of the model are only 916,594 and 1.4 GFLOPs, and the model achieves an average accuracy of 98.48%. Compared with the benchmark model YOLOv8n-cls model, the number of parameters and GFLOPs decreased by 36.6% and 58.8%, and the average accuracy improves by 2.96%. The standard deviation of the experimental data shows a decrease from 0.89% in the baseline model to 0.38%, and the stability of the model is also significantly enhanced.

This result fully proves the scientific nature of the synergistic optimization of the three modules (CBAM_ADown, C3ghost, and AFC_SPPF), rather than a simple superposition of the modules. The feature purification capability of CBAM_ADown lays the foundation for the model’s accurate recognition. The lightweight design of C3ghost reduces computational overheads while guaranteeing feature delivery efficiency, and generates more fine-grained information. The multi-scale aggregation capability of AFC_SPPF further makes up for the shortcomings of feature expression in complex scenes, ultimately forming a double breakthrough in “accuracy improvement” and “lightweight”. For the sunflower identification task under a complex field background, YOLO-CGA significantly reduces the deployment threshold of edge devices. At the same time, it can more robustly cope with the actual scene where the scale of the disease varies significantly and there are many background interferences. This provides efficient support for the rapid detection and accurate prevention and control of diseases in the field.

### 3.2 Comparative experiments on different attention mechanisms

We introduce the CBAM attention mechanism into the ADown module, which enhances the model’s ability to extract key features and its resistance to complex backgrounds. To validate the effectiveness of the ADown module combined with different attention mechanisms in improving the model performance, and to explore its ability to balance the lightweight property with classification accuracy, we design comparative experiments. We select a variety of mainstream attention mechanisms, such as Squeeze and Excitation (SE) (Hu et al., 2018), Efficient Channel Attention (ECA) (Wang et al., 2020), Spatial Intersection Attention Module (SiAM) (Han et al., 2024), and Global Attention Mechanism (GAM) (Liu et al., 2021), each of which has its own advantages in key feature extraction and background noise suppression. **Table 4** presents a comparison of the number of parameters of the modules (ADown combined with each attention mechanism) for different channel configurations.

**Table 4:** Number of parameters of the modules.

Channel	Parameters					
	ADown	SE_ADown	ECA_ADown	SiAM_ADown	CBAM_ADown	GAM_ADown
164→128	20,736	21,760	20,742	20,736	21,956	226,176
128→256	82,432	86,528	82,442	82,432	86,724	902,912
256→512	328,704	345,088	328,714	328,704	345,284	3,608,064

We use YOLOv8n-cls as a baseline model to design a series of comparative experiments, in which we either only introduce the ADown module or introduce the ADown module combined with the SE, ECA, SiAM, CBAM, and GAM attention mechanisms, respectively. As shown in **Table 5**, from the results of these comparative experiments, the combination of the ADown module and attention mechanisms can stably improve the model performance. For the YOLO_ADown model, which only incorporates the ADown module and is based on the original YOLOv8n-cls model, the average accuracy has been improved to 97.14%. When the ADown module is further combined with the attention mechanisms (e.g., SE, ECA, SiAM, CBAM, and GAM), the average accuracies of all the derived models have been improved to varying degrees. Among them, the model with ADown combined with GAM achieves the highest accuracy, but the number of parameters is significant. In contrast, the model with ADown combined with CBAM, strikes a better balance between lightweight property and accuracy.

**Table 5:** Results of comparative experiments on different attention mechanisms.

Model	Parameters	GFLOPs	Average accuracy ± standard deviation (%)
YOLOv8n-cls	1,445,974	3.4	95.52 ± 0.89
YOLO_ADown	1,163,094	2.7	97.14 ± 0.35
YOLO_SiAM_ADown	1,168,782	2.7	97.90 ± 0.20
YOLO_SE_ADown	1,174,170	2.7	97.68 ± 0.49
YOLO_ECA_ADown	1,163,992	2.7	97.88 ± 0.48
YOLO_CBAM_ADown	1,169,378	2.7	97.95 ± 0.20
YOLO_GAM_ADown	2,257,838	5.4	97.98 ± 0.35

In summary, the combination of the ADown module and attention mechanism shows strong versatility and scalability. Notably, the integration of the CBAM_ADown module strikes an effective balance between lightweight and accuracy. This balance provides a new way of thinking for feature purification in complex contexts.

### 3.3 AFC_SPPF module trunk branch K-value sensitivity and location adaptation

To optimize the feature fusion effect of the AFC_SPPF module, this section explores its core parameters and deployment strategy through two sets of experiments. First, we analyze the impact of different main-branch pooling kernel sizes (K = 3, 5, 7, 9) on model performance, and clarify the optimal values for adapting to agricultural scenarios. The second set of experiments is to validate the effectiveness of the module’s deployment at different network locations and identify efficient locations for feature fusion. All experiments were carried out on the BARI-Sunflower dataset, based on the YOLO-CGA model architecture (with integrated CBAM_ADown and C3ghost modules).

As shown in **Table 6**, the experimental results showed that when K = 3, the receptive field of the primary and auxiliary branches (3×3) had a high degree of overlap. This high overlap led to feature redundancy. At the same time, it also weakened the ability to capture the global features of sunflower disease identification. When K = 5, the main branch and the two auxiliary branch form “3×3 (detail) + 5×5 (medium) + 5×5×5 (global)” complementary feature structure. When combined with the original feature identification branch, this structure preserved the edge details of lesions while covering the overall characteristics of the disease, achieving an average accuracy as high as 98.48%. When K = 7 or K = 9, the receptive field of the trunk branch was too large. This excessively large receptive field causes excessive smoothing, which led to the loss of local fine-grained features. This feature loss further results in a decrease in feature fusion efficiency and a drop in accuracy.

**Table 6:** Results of comparison experiments on different K values of the trunk branches of the AFC_SPPF module.

K	Average accuracy ± standard deviation (%)
3	98.16 ± 0.41
5	98.48 ± 0.38
7	98.25 ± 0.35
9	97.91 ± 0.43

In addition, to explore the optimal deployment location of AFC_SPPF, we take the K value of the main branch to be 5, and deploy it in different C3ghost modules, as shown in ①, ②, ③, and ④ in **Figure 4(b)**, respectively. The experimental results are presented in **Table 7**. The model achieves the highest accuracy when the AFC_SPPF module is deployed after the last C3ghost module. This is because the output feature of the last C3ghost module has completed high-level semantic aggregation. At this point, the multi-scale fusion of AFC_SPPF can directly optimize the key features used for classification. And this optimization is achieved with the highest aggregation efficiency.

**Table 7:** Results of comparative experiments on the AFC_SPPF module at different locations.

Locations	Parameters	GFLOPs	Average accuracy ± standard deviation (%)
①	655,634	1.4	97.81 ± 0.46
②	668,338	1.4	98.08 ± 0.35
③	718,322	1.4	98.32 ± 0.21
④	916,594	1.4	98.48 ± 0.38

In summary, for the AFC_SPPF module, the model achieves optimal performance when the main trunk branch K-value is set to 5. It can achieve this optimal performance because the module balances lesion spot details and global features through a multi-scale complementary structure. This balance between details and global features effectively avoids two key issues: feature redundancy and the loss of fine-grained features. Separately, the model reaches the highest accuracy when the AFC_SPPF module is deployed after the last C3ghost module, because high-level semantic feature aggregation at this stage enables direct optimization of key features for classification and thus improves fusion efficiency.

### 3.4 Generalization experiments

To validate the effectiveness and superiority of our proposed model (YOLO-CGA) in the sunflower disease identification task, we designed comparative experiments. These experiments were conducted using the BARI-Sunflower dataset, where we compared our YOLO-CGA model with other mainstream lightweight models and evaluated its performance. The experimental results are shown in **Table 8**.

**Table 8:** Results of comparative experiments on different models on the BARI-Sunflower dataset.

Model	Parameters	GFLOPs	Average accuracy ± standard deviation (%)
EfficientNetV2-Small	20,185,174	14.9	92.86 ± 0.92
GhostNetV2_0.5	4,883,594	0.9	87.27 ± 0.79
MobileNetV3-Small	1,524,006	0.3	91.79 ± 0.69
YOLOv8n-cls	1,445,974	3.4	95.52 ± 0.89
MobileViT-XXS	952,950	1.3	88.05 ± 0.97
SqueezeNet1.0	738,502	4.0	91.11 ± 1.07
ShuffleNetV2 (1×)	1,259,754	0.8	88.49 ± 0.63
YOLOv10n-cls	1,536,854	3.5	98.01 ± 0.38
YOLOv11n-cls	1,538,790	3.3	96.03 ± 0.54
YOLOv12n-cls	1,726,182	3.7	97.71 ± 0.75
YOLOv13n-cls	1,581,458	3.3	97.95 ± 0.42
YOLO-CGA (ours)	916,594	1.4	98.48 ± 0.38

The experimental results show that the YOLO-CGA model achieves the triple advantages of “high accuracy, light weight, and high stability” in the task of sunflower disease identification under complex field background. The YOLO-CGA model achieves the highest accuracy among all compared models, reaching 98.48%. Its performance exceeds that of mainstream lightweight models, such as MobileNetV3-Small and GhostNetV2_0.5, by a substantial margin of 6.69% to 11.21%. Furthermore, it even outperforms EfficientNetV3-Small, despite the latter’s considerably larger parameter count and computational requirements. The YOLO-CGA model achieves an extreme lightweight design, with approximately the number of 916,600 parameters and 1.4 GFLOPs. Compared to other models in the same YOLO series, the number of parameters is reduced by 36.6% to 46.9%, while its computational efficiency significantly outperforms most comparative models. Specifically, the model demonstrates excellent stability, evidenced by a low accuracy standard deviation of 0.38, making it particularly powerful for complex field scenarios with enhanced anti-interference capability.

**Figure 8:**
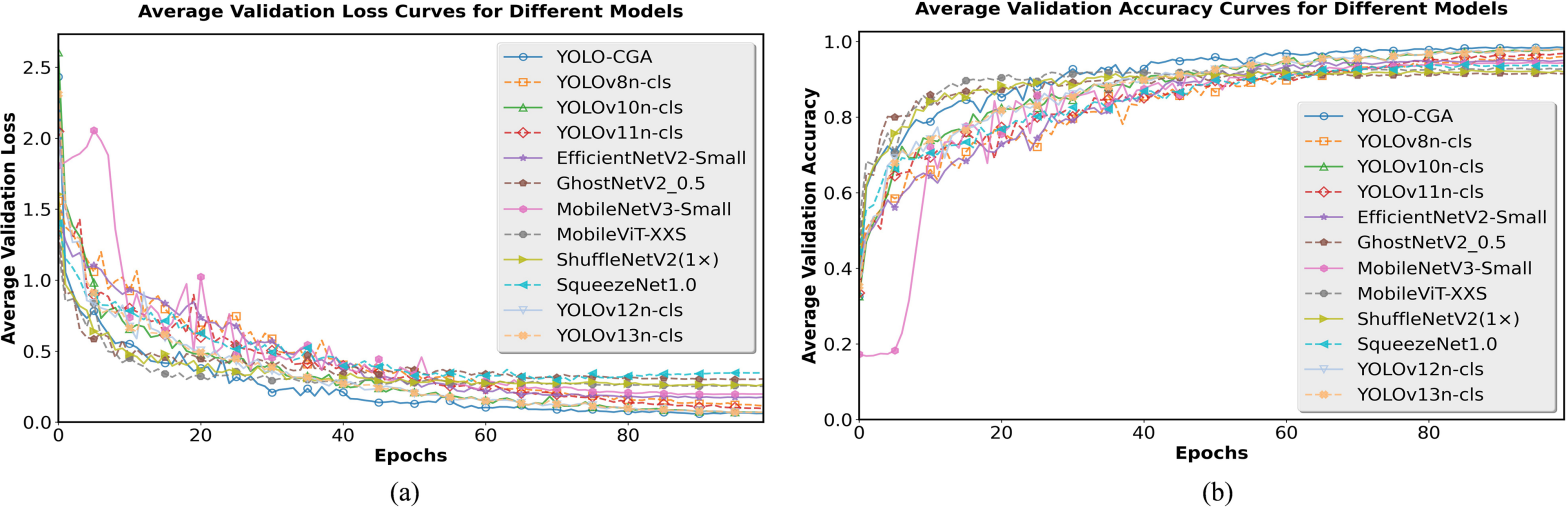
Average verification loss curve **(a)** and Average verification accuracy curve **(b)**.

The default training process of YOLO series model does not record the accuracy of each round of training round. Therefore, we only present the loss curve and accuracy curve of during the validation process to observe the model’s performance changes in this stage. We first calculated the average of the validation loss and validation accuracy for each fold in each round. Then we used these computed averages to plot the average validation loss curve and the average validation accuracy curve. As shown in **Figure 8(a)**, in terms of average validation loss, the YOLO-CGA model’s loss value decreases more rapidly and eventually converges to a lower level. As shown in **Figure 8(b)**, in the average validation accuracy curve, the YOLO-CGA model is the first to exceed the high accuracy threshold and maintains this high accuracy stably thereafter.

**Figure 9:**
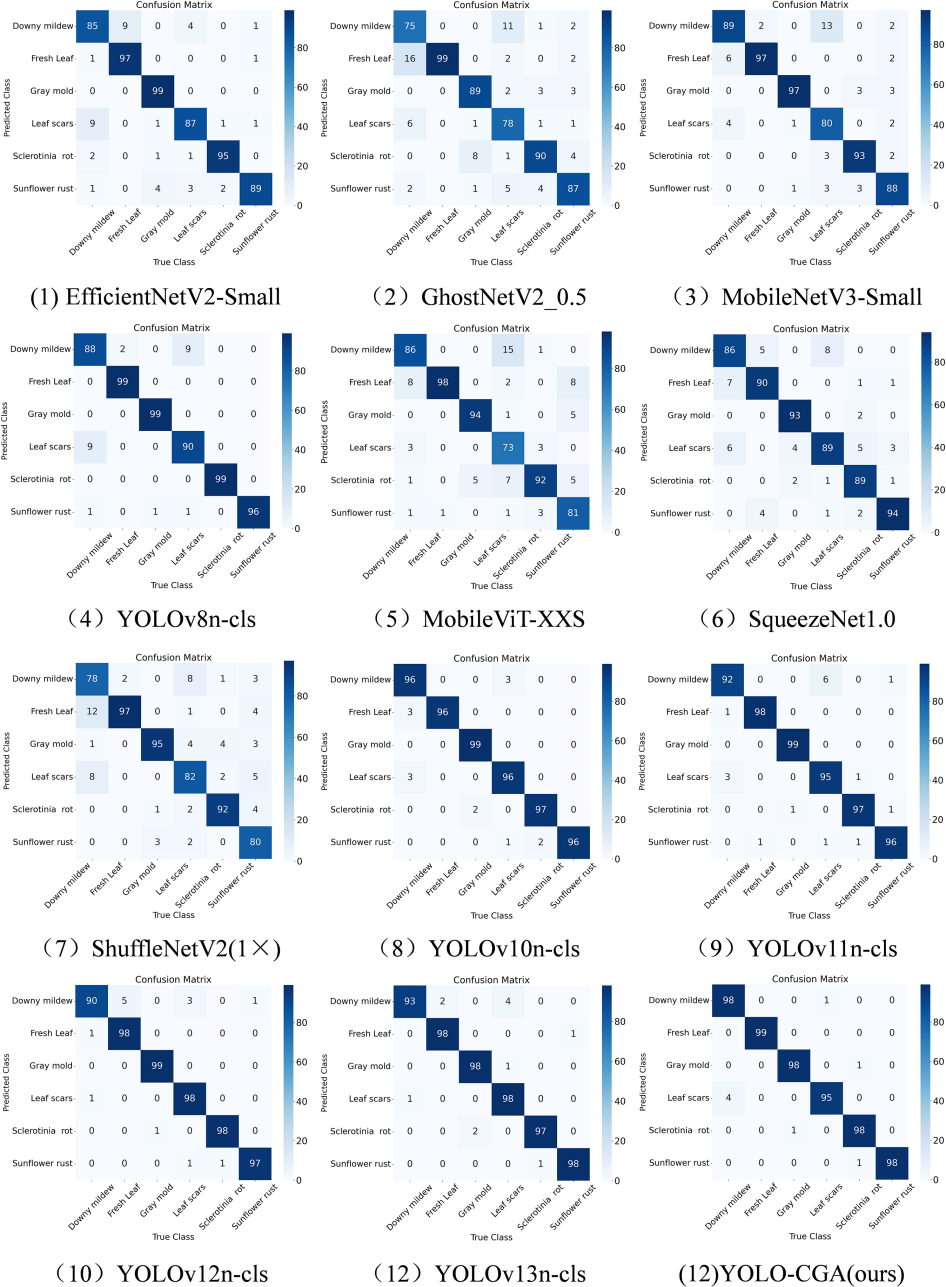
Confusion matrix for the different models.

To evaluate the classification performance of each model across different categories, this study selects the confusion matrix corresponding to the fold with the test accuracy closest to the mean among all folds in the cross-validation experiment for analysis, as shown in **Figure 9**. Meanwhile, the mean values of recall, precision, and F1-score across all folds are adopted as the core performance metrics for comparison, as presented in **Table 9**. Analysis based on the confusion matrix and category-specific performance metrics (recall, precision, and F1-score) demonstrates that the YOLO-CGA model exhibits outstanding and stable classification performance across all six plant disease categories. Compared with other models, YOLO-CGA achieves F1-scores higher than or comparable to the state-of-the-art across all categories (e.g., 97.20 ± 1.95 for Downy mildew and 98.20 ± 0.45 for Sunflower rust). Additionally, the small standard deviations of its recall and precision indicate high consistency in model predictions. Particularly in the Fresh Leaf and Gray mold categories, the recall of YOLO-CGA approaches 100%, which is significantly superior to other YOLO variants (such as YOLOv10n-cls and YOLOv13n-cls) and lightweight models (such as MobileNetV3-Small). Overall, while maintaining lightweight model, YOLO-CGA achieves comprehensive improvements in classification accuracy, verifying its effectiveness in this task.

**Table 9:** Comparison of classification performance metrics of various models across different sunflower disease categories.

Model	Class	Average recall ± standard deviation (%)	Average precision ± standard deviation (%)	Average F1-score ± standard deviation (%)
EfficientNetV2-Small	Downy mildew	87.60 ± 4.04	88.80 ± 2.39	88.00 ± 2.12
	Fresh leaf	98.20 ± 0.84	91.20 ± 2.77	94.80 ± 2.12
	Gray mold	98.00 ± 1.41	93.80 ± 1.92	96.00 ± 0.89
	Leaf scars	88.00 ± 1.87	94.00 ± 1.73	90.80 ± 1.30
	Sclerotinia rot	96.20 ± 1.79	94.40 ± 3.05	95.20 ± 2.28
	Sunflower rust	90.00 ± 2.00	95.60 ± 2.30	92.60 ± 1.94
GhostNetV2_0.5	Downy mildew	80.40 ± 3.05	83.80 ± 2.95	82.00 ± 1.83
	Fresh leaf	95.80 ± 3.03	87.00 ± 3.16	91.00 ± 1.22
	Gray mold	95.00 ± 2.97	89.80 ± 2.77	92.20 ± 1.30
	Leaf scars	79.60 ± 1.52	85.60 ± 3.05	82.40 ± 1.14
	Sclerotinia rot	88.40 ± 4.60	89.00 ± 2.74	88.60 ± 1.94
	Sunflower rust	85.20 ± 2.59	89.00 ± 5.70	87.00 ± 3.00
MobileNetV3-Small	Downy mildew	88.40 ± 6.51	85.60 ± 3.92	86.80 ± 1.67
	Fresh leaf	98.40 ± 0.80	90.20 ± 1.30	94.20 ± 0.84
	Gray mold	97.80 ± 1.10	94.60 ± 2.30	96.20 ± 1.30
	Leaf scars	83.80 ± 4.92	92.00 ± 2.45	87.60 ± 1.82
	Sclerotinia rot	94.80 ± 0.75	93.20 ± 2.17	94.00 ± 0.79
	Sunflower rust	88.00 ± 1.79	96.60 ± 2.41	92.00 ± 1.22
YOLOv8n-cls	Downy mildew	90.60 ± 2.07	89.60 ± 2.07	90.00 ± 1.58
	Fresh leaf	98.80 ± 1.10	98.00 ± 1.41	98.20 ± 0.45
	Gray mold	99.80 ± 0.45	96.80 ± 1.79	98.20 ± 0.45
	Leaf scars	90.80 ± 1.10	92.40 ± 2.61	91.40 ± 1.52
	Sclerotinia rot	97.60 ± 1.67	98.40 ± 1.30	97.80 ± 1.10
	Sunflower rust	96.00 ± 1.58	98.60 ± 1.67	96.80 ± 1.30
MobileViT-XXS	Downy mildew	79.80 ± 5.20	86.00 ± 5.10	82.40 ± 3.61
	Fresh leaf	97.60 ± 2.19	82.60 ± 4.32	89.40 ± 3.13
	Gray mold	95.80 ± 2.04	92.00 ± 2.61	93.80 ± 1.30
	Leaf scars	79.00 ± 2.97	87.60 ± 4.04	83.00 ± 0.98
	Sclerotinia rot	92.00 ± 1.83	87.00 ± 2.24	89.40 ± 1.52
	Sunflower rust	84.80 ± 2.68	94.60 ± 3.92	89.20 ± 1.48
SqueezeNet1.0	Downy mildew	85.00 ± 3.32	86.80 ± 1.30	85.80 ± 1.64
	Fresh leaf	95.20 ± 2.28	93.60 ± 2.07	94.60 ± 2.07
	Gray mold	94.60 ± 2.67	96.60 ± 1.34	95.40 ± 1.95
	Leaf scars	88.40 ± 2.41	85.00 ± 5.34	86.40 ± 2.41
	Sclerotinia rot	92.40 ± 2.19	93.20 ± 2.17	92.60 ± 0.89
	Sunflower rust	91.40 ± 2.60	92.30 ± 2.41	91.80 ± 1.79
ShuffleNetV2 (1×)	Downy mildew	78.60 ± 5.01	89.00 ± 2.24	83.40 ± 2.60
	Fresh leaf	97.00 ± 1.83	88.80 ± 2.59	92.80 ± 1.67
	Gray mold	94.60 ± 1.95	90.20 ± 1.79	92.20 ± 1.52
	Leaf scars	82.60 ± 4.60	83.80 ± 6.51	84.00 ± 2.97
	Sclerotinia rot	92.20 ± 3.40	87.20 ± 3.63	89.60 ± 1.82
	Sunflower rust	84.80 ± 3.63	93.40 ± 2.61	88.60 ± 1.52
YOLOv10n-cls	Downy mildew	96.20 ± 2.49	96.40 ± 1.95	96.20 ± 1.48
	Fresh leaf	99.40 ± 1.10	99.80 ± 0.45	99.40 ± 0.80
	Gray mold	99.80 ± 0.45	99.20 ± 0.84	99.40 ± 0.45
	Leaf scars	96.00 ± 0.89	95.80 ± 2.49	95.60 ± 1.14
	Sclerotinia rot	98.80 ± 0.80	98.20 ± 0.45	98.20 ± 0.45
	Sunflower rust	98.00 ± 0.89	99.00 ± 1.22	95.42 ± 0.49
YOLOv11n-cls	Downy mildew	89.40 ± 2.41	94.20 ± 2.49	91.40 ± 1.52
	Fresh leaf	98.80 ± 0.45	97.60 ± 1.30	98.00 ± 0.75
	Gray mold	99.20 ± 0.45	98.80 ± 0.80	99.00 ± 0.45
	Leaf scars	95.20 ± 2.28	90.60 ± 1.95	92.80 ± 1.82
	Sclerotinia rot	97.20 ± 1.48	98.40 ± 1.10	97.60 ± 0.49
	Sunflower rust	97.60 ± 0.80	98.20 ± 1.10	97.60 ± 0.49
YOLOv12n-cls	Downy mildew	94.20 ± 3.36	97.60 ± 1.52	95.20 ± 1.30
	Fresh leaf	99.20 ± 0.45	97.20 ± 2.49	98.00 ± 0.89
	Gray mold	99.60 ± 0.49	98.40 ± 1.10	98.80 ± 0.45
	Leaf scars	98.00 ± 1.41	96.20 ± 2.61	97.20 ± 1.10
	Sclerotinia rot	97.80 ± 1.10	98.60 ± 0.49	98.00 ± 0.80
	Sunflower rust	97.80 ± 1.10	98.20 ± 0.45	97.80 ± 0.80
YOLOv13n-cls	Downy mildew	95.80 ± 2.87	97.20 ± 1.73	96.50 ± 1.36
	Fresh leaf	99.50 ± 0.52	98.50 ± 1.17	99.00 ± 0.68
	Gray mold	99.70 ± 0.41	99.00 ± 0.76	99.30 ± 0.40
	Leaf scars	97.50 ± 1.29	96.80 ± 2.15	97.10 ± 1.02
	Sclerotinia rot	98.50 ± 0.95	98.80 ± 0.63	98.60 ± 0.57
	Sunflower rust	98.20 ± 0.98	98.60 ± 0.72	98.40 ± 0.61
YOLO-CGA (ours)	Downy mildew	97.80 ± 2.07	96.60 ± 2.07	97.20 ± 1.95
	Fresh leaf	99.80 ± 0.45	98.80 ± 1.30	99.20 ± 0.75
	Gray mold	99.20 ± 0.80	99.20 ± 0.80	99.00 ± 0.45
	Leaf scars	97.20 ± 1.48	97.40 ± 1.52	97.00 ± 1.41
	Sclerotinia rot	99.20 ± 0.80	99.00 ± 0.89	98.60 ± 0.49
	Sunflower rust	97.60 ± 1.30	99.80 ± 0.45	98.20 ± 0.45

To verify the generalization ability of YOLO-CGA on different crop datasets, we conducted experiments on two cross-crop datasets, namely the Cotton disease dataset and FGVC8. The experimental results presented in **Table 10**. YOLO-CGA achieved optimal on both public datasets. The average accuracy on the Cotton disease dataset reaches 98.32%, representing a 0.14% improvement over the benchmark YOLOv8n-cls model. On the FGVC8 dataset reaches, the accuracy reached 91.11%, which is a 2.85% improvement over the baseline YOLOv8n-cls model. This superior performance can be attributed to the synergistic effects of three key components: CBAM_ADown (for suppressing background noise), AFC_SPPF (for adapting to disease scale variations), and C3ghost (for generating fine-grained features). Collectively, these modules enable the proposed YOLO-CGA model to perform excellently not only in sunflower disease recognition but also in cross-crop and diverse disease scenarios. This thus demonstrates the model’s outstanding generalization capability.

**Table 10:** Results of generalization experiments on two public cross-crop datasets.

Model	Average accuracy ± standard deviation (%)	
	Cotton disease dataset	FGVC8
EfficientNetV2-Small	96.12 ± 0.31	85.75 ± 0.37
GhostNetV2_0.5	92.04 ± 1.23	85.54 ± 0.39
MobileNetV3-Small	95.12 ± 0.57	84.58 ± 0.45
YOLOv8n-cls	98.18 ± 0.40	88.26 ± 0.24
MobileViT-XXS	92.12 ± 0.61	84.92 ± 0.56
SqueezeNet1.0	95.78 ± 0.50	84.98 ± 0.23
ShuffleNetV2 (1×)	94.22 ± 0.83	85.10 ± 0.31
YOLOv10n-cls	98.05 ± 0.22	89.08 ± 0.18
YOLOv11n-cls	98.22 ± 0.21	88.36 ± 0.20
YOLOv12n-cls	98.20 ± 0.44	88.81 ± 0.21
YOLOv13n-cls	98.28 ± 0.25	89.63 ± 0.19
YOLO-CGA (ours)	98.32 ± 0.20	91.11 ± 0.23

### 3.5 Edge device deployment

We expect that the proposed YOLO-CGA model will not only remain at the theoretical level of performance improvement, but also pay more attention to the translation to practical application scenarios. The Raspberry Pi offers significant advantages, including low cost, compact size, and low power consumption. With adequate computing capability to support real-time inference of lightweight models, it is highly suitable for deployment in edge computing scenarios and widely used in intelligent applications such as agricultural robots and drones.

**Figure 10:**
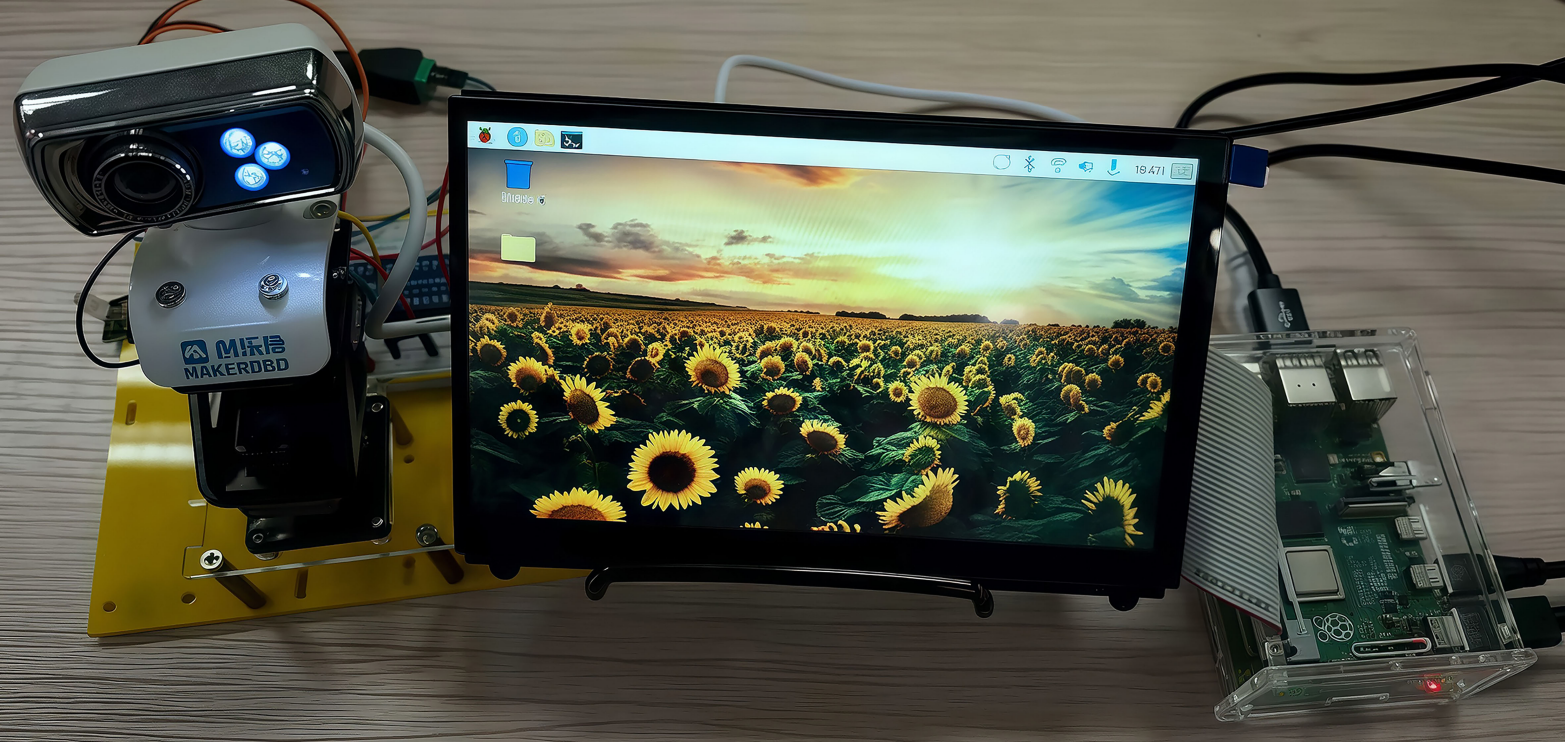
Physical photograph of the raspberry Pi 4B edge deployment.

The Raspberry Pi 4B development board was selected for this study, and its key configurations mainly include: a Broadcom BCM2711 quad-core Cortex-A72 processor (1.5GHz), 4GB of LPDDR4 RAM, an original camera, an MIPI CSI camera interface, an MIPI DSI display interface, and 7-inch touchscreen display with a resolution of 1024×600. A physical diagram of the Raspberry Pi 4B edge deployment is shown in **Figure 10**.

The system’s GUI interface is developed using PyQt5. The sunflower disease recognition system supports two modes: image loading and recognition, taking pictures for recognition. For the image loading and recognition mode, local images can be imported directly to complete the recognition process. For the image capture and recognition mode, the system automatically turns on the camera; after the user clicks the capture button, the photo is saved, and this triggers the model to load and output the recognition results.

For the deployment of the model in this study, the conversion workflow of “PyTorch, ONNX, TensorRT” is adopted, and the specific steps are as follows: First, as the step of Model Export, the trained YOLO-CGA PyTorch model is exported to the ONNX format (version 1.12) to ensure lossless conversion of the model structure and weights, and during export, the input dimension is specified as (1, 3, 512, 512) to match the hardware input requirements of the Raspberry Pi; then, in the step of Quantization Optimization, to further reduce model inference latency and memory usage, the INT8 quantization method provided by TensorRT is used to optimize the ONNX model, the validation set of the BARI-Sunflower dataset is employed as the calibration set during the quantization process, quantization parameters for the weights of each layer are obtained through calibration, and the model weights are converted from 32-bit floating-point (FP32) to 8-bit integer (INT8) under the premise that the model accuracy loss is controlled within 0.5%; finally, regarding the Deployment Framework, TensorRT 8.6 is used as the inference framework on the Raspberry Pi 4B, a GUI interactive interface is developed with PyQt5 to implement two functions: image loading and recognition, and real-time recognition via photographing, and TensorRT further improves the inference efficiency of the model on the ARM architecture through technologies such as layer fusion and automatic kernel optimization.

**Figure 11:**
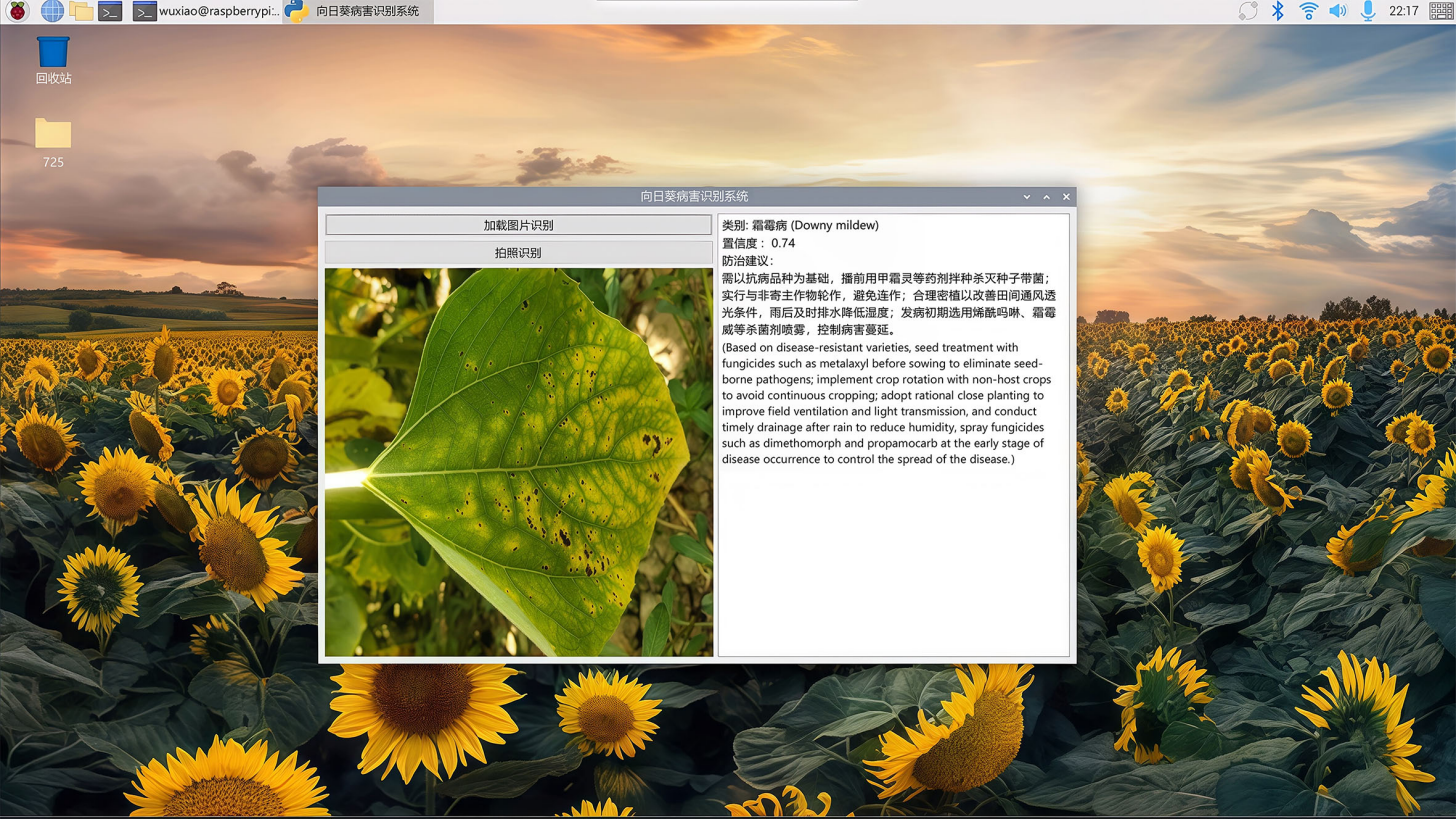
Test rendering of sunflower disease identification system.

To verify the deployment effectiveness, we conducted field tests by placing the Raspberry Pi edge device in the field, successfully achieving accurate identification of sunflower diseases. The test results are shown in **Figure 11**. Meanwhile, to quantify the model deployment performance, **Table 11** presents the core performance metrics of the YOLO-CGA model on the Raspberry Pi 4B.

**Table 11:** Deployment performance metrics of the YOLO-CGA model on raspberry Pi 4B.

Performance metrics	Value
Inference Time per Image (ms)	128.7 ± 3.5
Frame Rate (FPS)	7.8 ± 0.5
Memory Usage During Inference (RAM)	486 ± 22 MB
Device Operating Power Consumption (W)	3.7 ± 0.3

All performance metrics in **Table 11** were tested under actual field conditions on the Raspberry Pi 4B (4GB RAM version) with strictly unified test parameters: the system was Raspbian 11, the CPU frequency was 1.5GHz, the GPU was Broadcom VideoCore VI, and the test image resolution was consistent with the model input dimension (512×512). Among these metrics, the inference time per image represents the mean ± standard deviation of 100 consecutive inference runs, and the frame rate corresponds to the average number of images processed per second during the inference phase.

Test results indicate that the YOLO-CGA model achieves an inference frame rate of 7.8 ± 0.5 FPS on the Raspberry Pi, which is significantly higher than the minimum requirement for real-time field detection (generally ≥ 5 FPS). The inference time per image is 128.7 ± 3.5 ms, the memory usage is controlled within 500 MB, and the device’s operating power consumption is 3.7 ± 0.3 W. This enables the Raspberry Pi to operate continuously for extended periods using a portable power bank, fully adapting to the practical application scenario where there is no external power supply in the field.

## 4 Discussion

This study addresses the need for lightweight models and highly robust recognition of sunflower diseases in complex field environments. To this end, we propose the YOLO-CGA model, which incorporates synergistic feature purification, fine-grained generation, and adaptive fusion strategy. The effectiveness of the proposed model is demonstrated via a series of systematic experiments.

The experimental results show that the core innovation module of the YOLO-CGA model realize the synergistic optimization of accuracy, lightweight performance, and robustness. By integrating an attention mechanism with a two-branch downsampling structure, the CBAM_ADown module effectively suppresses complex background interference (e.g., soil and weeds) while preserving key disease features. This design improves the model’s accuracy by 2.43%. The result also validates the effectiveness of the “attentional downsampling” paradigm for feature purification in agricultural scenarios. The C3ghost module employs Ghost convolution’s cost-effective to generate more fine-grained features. It reduces the number of parameters by 35.84% while maintaining baseline performance stability. Furthermore, it effectively resolves the computational redundancy issue inherent in the traditional C2f module. The multi-branch adaptive fusion mechanism in the AFC_SPPF module accurately captures multi-scale disease features. When being synergized with the first two modules, it further increases the model’s accuracy to 98.48%. This result proves the strong adaptability of the feature purification, fine-grained generation, and multi-scale fusion architecture in handling complex field environments.

**Figure 12:**
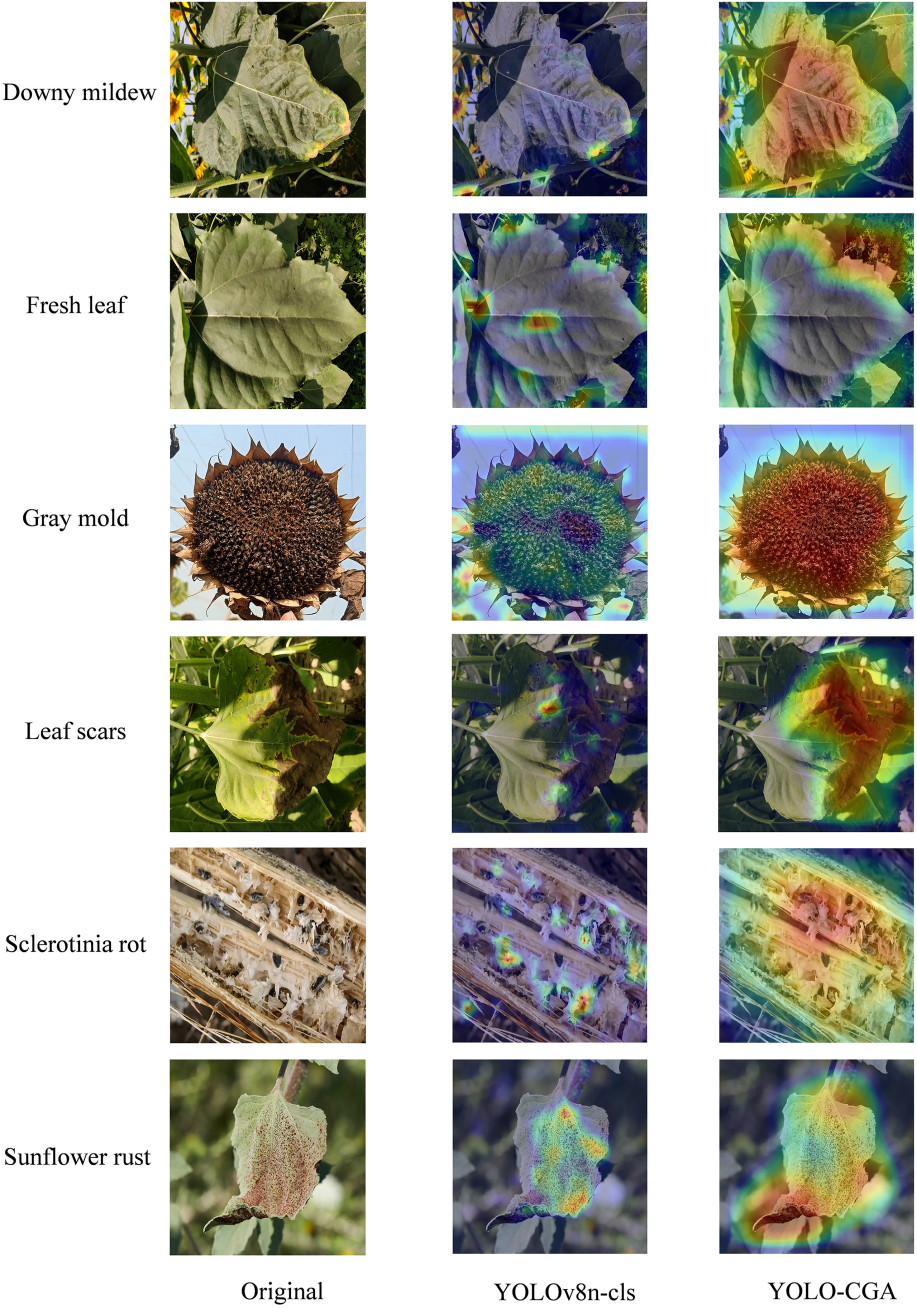
Activation heatmaps of the YOLOv8n-cls and YOLO-CGA models.

As shown in **Figure 12**, we visualized the activation heatmaps of the YOLO-CGA model and the baseline model YOLOv8n-cls for different disease categories in the final layer. A detailed analysis of the generated heat maps reveals that the YOLO-CGA model is more accurately focused on the diseased areas. In Downy mildew, Gray mold and other disease samples, its thermal distribution better fits the lesion contour, with clear lesion boundaries and less background interference. For healthy samples such as Fresh leaf, it can also accurately anchor the effective area of the leaf. In contrast, YOLOv8n-cls thermal often shows “diffusion blurring” or “localization offset”, reflecting that YOLO-CGA is more advantageous in capturing fine-grained features, and more accurate in spatial localization and feature differentiation of the disease.

Some recent studies’ models for these three datasets are compared with our proposed model, as shown in **Table 12**. On the BARI-Sunflower dataset, the parameter sizes of the existing models vary widely, ranging from 0.03M (TeenyNet) to 22M (EfficientNetV2-Small). Although EfficientNetV2-Small achieves the highest performance at 99.2%, it comes at the cost of a significantly larger model size. YOLO-CGA achieves 98.48% accuracy in the six-category classification task, while maintaining a notably higher ability to handle complexity in the four-category task compared to other models. With the number of parameters being only 0.92M, it demonstrates an outstanding balance among the number of target categories, parameter efficiency, and overall performance. For the Cotton Disease Dataset, Vision Transformer achieved 98.50% accuracy, but the number of its parameters was not specified. The YOLO-CGA model achieves 98.32% performance with a parameter scale of 0.92M, which is close to the existing optimal results and maintains excellent classification accuracy among the lightweight model. On the FGVC8 dataset, the parameter sizes of existing models range from 1.76M to 28.2M. The best-performing model, DFN-PSAN, achieves the highest accuracy of 93.55% with 1.76M parameters. In comparison, the YOLO-CGA model attains 91.11% accuracy using only 0.92M parameters. Although slightly less accurate than DFN-PSAN, YOLO-CGA demonstrates significantly higher parameter efficiency while still maintaining competitive classification performance. In summary, YOLO-CGA is designed with a lightweight parameter size of only 0.92M and demonstrates strong generalization across multi-category tasks on diverse datasets. These results highlight its outstanding balance between model lightness and recognition accuracy.

**Table 12:** Models from recent relevant studies on the three datasets.

Dataset	Model	Parameters	Classes	Accuracy (%)
BARI-Sunflower	DFN-PSAN (Dai et al., 2024)	1.76M	4	94.23
	TeenyNet (Zhong and Tong, 2024)	0.03M	4	98.94
	EfficientNetV2-Small (Zhang and Wu, 2025)	22M	4	99.2
	VGG19+CNN (Ghosh et al., 2023)	2.07M	4	93
	EfficientNetB3 (Gulzar et al., 2023)	–	4	97.9
	SunNet (Sathi et al., 2023)	–	4	97.88
	YOLO-CGA (ours)	0.92M	6	98.48
Cotton Disease Dataset	Vision Transformer (Zhao et al., 2023)	–	5	98.50
	YOLO-CGA (ours)	0.92M	5	98.32
FGVC8	EDIT (Feng et al., 2024)	5.6M	12	91.5
	DFN-PSAN (Dai et al., 2024)	1.76M	12	93.55
	AFD-Net (Yadav et al., 2022)	28.2M	6	92.6
	YOLO-CGA (ours)	0.92M	12	91.11

The success of the Raspberry Pi edge deployment further validates the practical value of the model. Unlike large-scale models that rely on high-performance servers, YOLO-CGA enables sunflower disease identification on low-cost embedded devices. This capability provides smallholder farmers and grassroots agricultural departments with a practical and affordable diagnostic tool. Furthermore, it effectively bridges the gap between digital agricultural technologies and real-world field applications.

## 5 Conclusions

In this study, we proposed the YOLO-CGA model. Its aim is to address the need for lightweight and practical sunflower disease identification in complex natural field contexts. We drew the following conclusions through systematic experiments and comparative analysis: (1) Modular synergistic optimization effectively enhances model performance. The collaborative effect of the CBAM_ADown, C3ghost, and AFC_SPPF modules achieves an organic integration of feature purification, fine-grained feature generation, and multi-scale fusion. On the BARI-Sunflower dataset, the proposed model shows notable advantages compared to the baseline model: it reduces the number of parameters by 36.6% and GFLOPs by 58.8%. Meanwhile, it reaches an accuracy of 98.48%, successfully balancing lightweight design with high accuracy. (2) The model demonstrates excellent generalization capability and robustness. Cross-dataset experiments show that YOLO-CGA model achieves accuracies of 98.32% and 91.11% in the cotton and apple disease recognition tasks, respectively. These results are significantly superior to those of most comparable models, confirming its strong adaptability across various crops and complex field environments. (3) The model achieves practical field deployment through edge computing. It has been successfully deployed on the Raspberry Pi 4B devices, enabling both local photo analysis and real-time photographic recognition. This provides a low-cost solution for crop disease identification in resource-limited scenarios and promotes the adoption of deep learning technology in grassroots agricultural applications.

There are still some limitations in this study: although the dataset was extended to six categories, the sample size is still limited. A smaller sample size not only puts the model at risk of overfitting but also fails to cover all possible disease scenarios and environmental conditions. These limitations thus affect the robustness of the model in real-world applications. From the confusion matrix, Downy mildew and Leaf scars have similar disease characteristics, and the model is prone to confusing the characteristics of these two diseases. Although the inference speed of the model on the Raspberry Pi can meet basic practical needs, we are not fully satisfied with it, and there is still room for optimization in the future.

In the future, our research can be advanced in four aspects: First, we will expand the number of categories and samples in sunflower datasets to improve the model’s robustness. Second, we will further explore the subtle features of the diseases further to enhance the model’s recognition accuracy. Third, we will optimize the module structure by integrating Neural Architecture Search (NAS) to increase the model’s inference speed. Fourth, we will integrate more Internet of Things (IoT) devices. On this basis, we will build an integrated sunflower disease identification system, which has the functions of identification, early warning, prevention, and control. This system can further support the development of precision agriculture.

## Data Availability

The original contributions presented in the study are included in the article/supplementary material, further inquiries can be directed to the corresponding author/s.
